# The lactate response to a second bout of exercise is not reduced in a concurrent lower-limb exercise program

**DOI:** 10.1038/s41598-023-48670-9

**Published:** 2023-12-04

**Authors:** Hayato Tsukamoto, Tadashi Suga, Kento Dora, Takeshi Sugimoto, Keigo Tomoo, Tadao Isaka, Takeshi Hashimoto

**Affiliations:** 1 Faculty of Sport Sciences, Waseda University, 2-579-15 Mikajima, Tokorozawa, Saitama 359-1192 Japan; 2 Institute of Advanced Research for Sport and Health Science, Ritsumeikan University, Kusatsu, Shiga Japan; 3 Faculty of Sport and Health Science, Ritsumeikan University, Kusatsu, Shiga Japan; 4 Department of Biomedical Engineering, Toyo University, Kawagoe, Saitama Japan; 5 Department of Nutrition Science, Purdue University, West Lafayette, IN USA

**Keywords:** Metabolism, Biomarkers

## Abstract

We aimed to evaluate the blood lactate level in response to two bouts of exercise. First, we hypothesized that blood lactate elevation in response to moderate-intensity aerobic exercise (MIAE) would be lower at the end of the second bout of MIAE than the first bout of MIAE. In this context, we also hypothesized that lactate accumulation at the end of resistance exercise (RE) would be reduced if MIAE is performed before RE (*i.e.*, concurrent exercise; CE). If so, we hypothesized that the order of the CE (*i.e.*, RE + MIAE *vs.* MIAE + RE) influences blood lactate kinetics. To test the hypotheses, forty-three healthy men participated in three studies. In study 1, 20 men (age 21 ± 2 years) performed two bouts of a 20-min MIAE separated by a 20-min rest interval. In study 2, 11 men (age 22 ± 1 years) performed RE only and CE (MIAE + RE; AR_CE_) with a 20-min rest interval in a crossover design. In study 3, 12 men (age 21 ± 2 years) performed both CEs, which were AR_CE_ and RE + MIAE (RA_CE_), with a 20-min rest interval in a crossover design. We measured blood lactate before and at the end of each exercise session. In study 1, the blood lactate response to the second bout of MIAE was lower than that of the first bout (*P* < 0.001, *r* = 0.68). However, the blood lactate response to the AR_CE_ trial was not lower than the response to the RE trial in study 2 (*P* = 0.475, *r* = 0.22). The results of study 3 showed that the RA_CE_ and AR_CE_ trials induced a similar lactate response (MIAE *P* = 0.423, *r* = 0.28; RE *P* = 0.766, *d* = 0.03). These observations indicate that whereas lactate accumulation might be diminished by a second bout of MIAE, a different type of exercise (*i.e.*, aerobic/resistance) did not result in a diminished lactate accumulation in response to a second bout of exercise.

## Introduction

Many studies have demonstrated the health benefits of physical activity and strategic ‘exercise’^1–5^, and these include preventing muscle atrophy^2^, weight gain^3^, and cognitive decline^4^. Among these benefits, it is widely known that resistance exercise (RE) can effectively induce muscle hypertrophy, while cardiorespiratory aerobic exercise (AE) can reduce body fat due to an increase in energy expenditure^2^. Given these different merits of RE and AE, it seems reasonable that the combination of RE and AE (*called* concurrent exercise; CE^6^) is a beneficial strategy for maintaining and improving health^2^. Notably, in terms of brain health, both RE (*e.g.*, knee extensions) and AE (*e.g.*, cycling exercise) improve cognitive function^7, 8^, while a meta-analysis by Colcombe and Kramer^9^ reported that CE has a greater impact on cognitive improvement than AE alone.

One potential physiological mechanism for the positive impacts of exercise is the bioavailability of lactate, which is produced through anaerobic glycolysis from glucose/glycogen^5, 10–14^. Lactate serves as not only an energy substrate but also as a myokine at rest and an exerkine during exercise^15, 16^. During exercise, lactate is primarily secreted by white-glycolytic muscle fibers in an exercise intensity-dependent manner and is distributed to ‘consumers’, such as the brain, heart, and liver, in addition to red-oxidative muscle fibers^5, 13–17^. When the muscle glycogen content is progressively decreased during prolonged AE, the ability to accumulate lactate may be diminished (*i.e.*, a low blood lactate concentration)^18^. In addition, our previous studies revealed that blood lactate is lower in response to a second bout of high-intensity interval AE (HIIE) than in response to the first bout of HIIE even though two identical exercise sessions were performed (*i.e.*, the same intensity and duration)^13, 19^. Potentially, the first bout of HIIE may approach muscle glycogen depletion, thereby creating a small elevation in blood lactate during repeated exercise. For example, during a soccer game, blood lactate elevation is diminished during the second half compared with the first half and is likely due to a decrease in muscle glycogen^20^. Given the dose-dependent effect of lactate on some health factors^11, 21, 22^, the positive impact of the second bout of HIIE on health can be lower than for the first bout of HIIE^13, 19^.

In general, the recommended amount of moderate-intensity physical activity to maintain and improve health is above 150 min/wk, implying that MIAE for at least 30 min/day (5 days/wk) is needed^2^. Moreover, a recommendation from the American College of Sports Medicine and the American Heart Association suggests that the benefits of MIAE can be accumulated through multiple bouts of short-duration exercise^1, 2^, indicating that both one bout of AE (*i.e.*, continuous AE) and multiple bouts of AE (*i.e.*, repeated AE) are suitable strategies for promoting health^2^. However, little is known about the blood lactate response to a second bout of MIAE (*i.e.*, around the lactate threshold). Similar to HIIE session (*i.e.*, above lactate threshold)^13, 19^, we hypothesized that the second bout of MIAE would result in reduced blood lactate elevation compared with the first bout. In this context, if the first bout of MIAE affects the blood lactate response to the second bout of exercise, we also hypothesized that blood lactate elevation in response to the RE trial would be reduced when the MIAE is performed before the RE. If so, it is possible the CE order influences blood lactate kinetics; namely, lactate accumulation in response to the CE program as MIAE + RE (AR_CE_) would be lower than that of RE + MIAE (RA_CE_). To advance our understanding of the bioavailability of lactate, we designed three studies that aimed to examine the blood lactate response to the second bout of exercise in two bouts of AE and CE programs. These findings would indicate a new idea to induce further lactate-enhancing accumulation in response to multiple bouts of exercise, which may contribute to health benefits.

## Results

### Study 1: The first bout versus the second bout of moderate-intensity aerobic exercise

The baseline heart rate (HR), blood glucose, and blood lactate were 67 ± 4 bpm, 97.7 ± 7.7 mg/dL, and 1.0 ± 0.1 mM, respectively.

**Figure 1:**
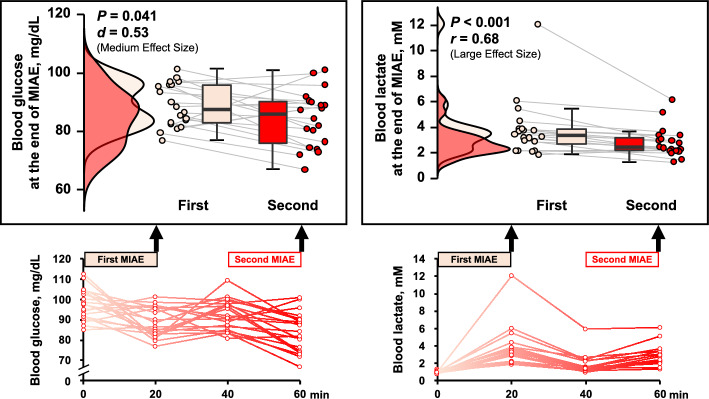
Blood glucose and lactate at the end of the first and second bouts of moderate-intensity aerobic exercise (MIAE) in study 1. The raincloud plots show the distribution of both glucose and lactate, circle plots represent individual data, and the box-and-whisker plots are median values (IQR and max/min).

The HR (*P* < 0.01, *d* = 0.46) and rating of perceived exertion (RPE) for leg effort (*P* < 0.05, *r* > 0.5) in response to the second bout of MIAE were higher than those in response to the first bout of MIAE (Table 1). Compared with the first bout, lower blood glucose (*P* < 0.05, *d* = 0.53) and lactate (*P* < 0.001, *r* > 0.5) levels were observed at the end of the second bout of MIAE (Fig. 1).

**Table 1:** The heart rate (HR) and a rating of perceived exertion (RPE) in response to moderate-intensity aerobic exercise (MIAE) in study 1.

	First MIAE	Repeated MIAE	*P*-value	Effect size
HR, bpm	153 ± 12	158 ± 10	**0.009**	***d* = 0.46**
RPE, 6–20 scale	13.5 (13–15)	15 (13–15)	0.068	*r* = 0.41
RPE, CR10 scale	4.5 (4–6)	6 (4–7)	**0.012**	***r* = 0.56**

Values are mean ± SD or median (IQR).

Significant values are in bold.

### Study 2: Resistance exercise versus moderate-intensity aerobic exercise + resistance exercise

The baseline HR (RE 64 ± 7 bpm *vs.* AR_CE_ 67 ± 8 bpm, *P* = 0.058, *d* = 0.51), blood glucose (RE 88.5 (87–101) md/dL *vs.* AR_CE_ 95.5 (90–99.5) mg/dL, *P* = 0.059, *r* = 0.57), and blood lactate (RE 1.2 ± 0.1 mM *vs.* AR_CE_ 1.2 ± 0.3 mM, *P* = 0.334, *d* = 0.36) were similar between the RE and AR_CE_ trials.

**Figure 2:**
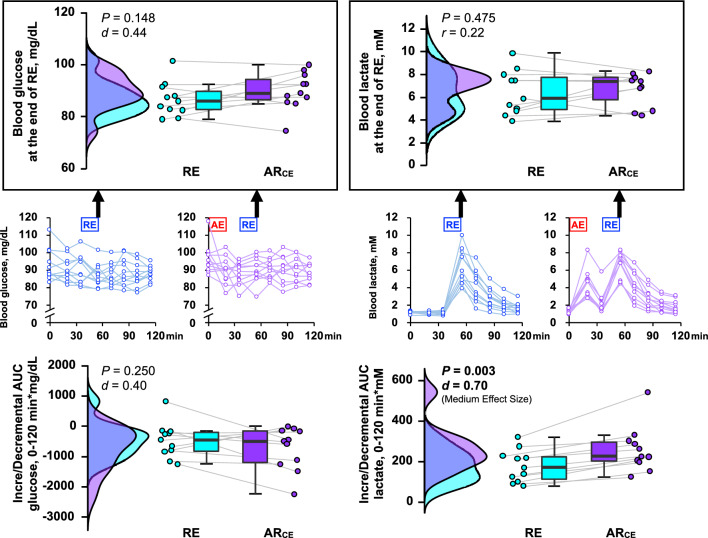
Blood glucose and lactate responses in study 2. The upper illustrations in the black boxes represent the data at the end of the resistance exercise (RE), while the lower illustrations represent the area under the curve (AUC) throughout the experiment. The raincloud plots show the distribution of both glucose and lactate. The circle plots represent individual data, and the box-and-whisker plots are median values (IQR and max/min). AE; aerobic exercise, AR_CE_; concurrent exercise (MIAE + RE).

Whereas the HR in response to RE in the AR_CE_ trial was higher than that in the RE trial (*P* < 0.05, *d* = 0.55), a similar RPE was observed in both trials (Table 2). Both glucose (*P* = 0.148, *d* = 0.44) and lactate (*P* = 0.475, *r* = 0.22) responses to RE were not different between the RE and AR_CE_ trials (Fig. 2). Additionally, the glucose area under the curve (AUC) throughout the RE trial was identical to that of the AR_CE_ trial (*P* = 0.250, *d* = 0.40). In contrast, the lactate AUC throughout the experiment was larger in the AR_CE_ trial than in the RE trial (*P* < 0.01, *d* = 0.70).

**Table 2:** The heart rate (HR) and a rating of perceived exertion (RPE) in response to the resistance exercise (RE) in study 2.

	RE	AR_CE_	*P*-value	Effect size
HR, bpm	121 ± 16	130 ± 15	**0.020**	***d* = 0.55**
RPE, 6–20 scale	17 (15–19)	17 (14–18)	0.564	*r* = 0.17
RPE, CR10 scale	9 (7–10)	9 (7–10)	1.000	*r* = 0.00

Values are mean ± SD or median (IQR). AR_CE_, concurrent exercise (MIAE + RE).

Significant values are in bold.

### Study 3: Moderate-intensity aerobic exercise + resistance exercise versus resistance exercise + moderate-intensity aerobic exercise

The baseline HR (RA_CE_ 67 ± 6 bpm *vs.* AR_CE_ 69 ± 5 bpm, *P* = 0.175, *d* = 0.42), blood glucose (RA_CE_ 91 ± 6 mg/dL *vs.* AR_CE_ 94 ± 8 mg/dL, *P* = 0.087, *d* = 0.44), and blood lactate (RA_CE_ 1.1 (1.0–1.5) mM *vs.* AR_CE_ 1.2 (1.1–1.4) mM, *P* = 0.326, *r* = 0.27) measurements were similar between the RA_CE_ and AR_CE_ trials.

**Figure 3:**
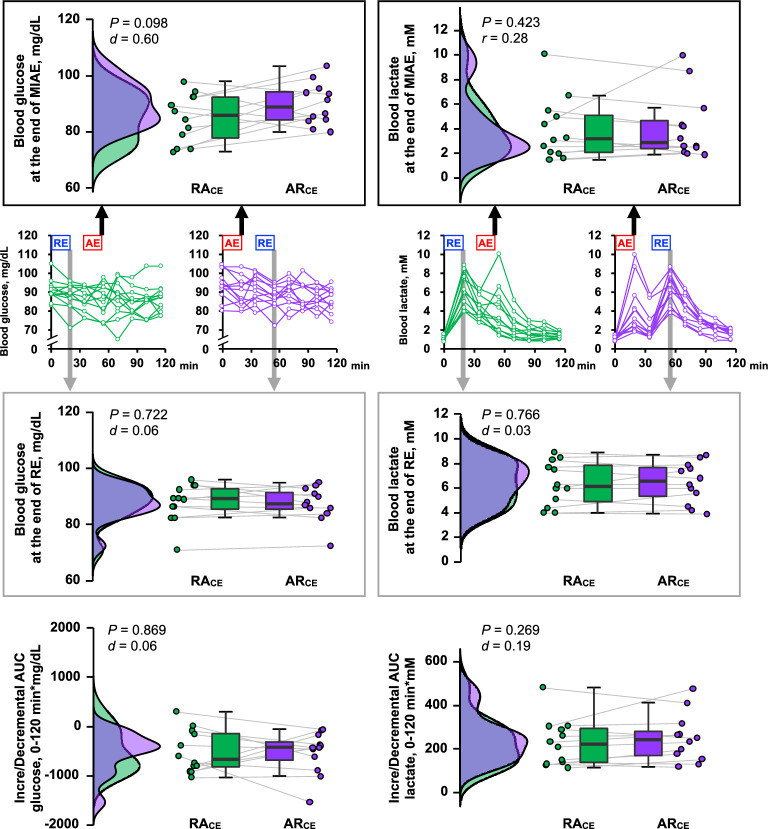
Blood glucose and lactate responses in study 3. The upper illustrations in the black boxes represent the data at the end of the MIAE, while the middle illustrations in gray boxes represent the data at the end of the RE. The lower illustrations represent the AUC throughout the experiment. The raincloud plots show the distribution of both glucose and lactate. The circle plots represent individual data, and the box-and-whisker plots are median values (IQR and max/min).

In the same way as in study 2, the RE-enhanced HR was higher in the AR_CE_ trial than in the RA_CE_ trial (*P* < 0.01, *d* = 0.54). Meanwhile, the HR response to MIAE was not different between the RA_CE_ and AR_CE_ trials (*P* = 0.455, *d* = 0.18). In terms of RPE, there were no differences between the RA_CE_ and AR_CE_ trials (Table 3). Additionally, blood glucose (*e.g.*, AUC RA_CE_ − 507 ± 436 min*mg/dL *vs.* AR_CE_ − 533 ± 429 min*mg/dL, *P* = 0.869, *d* = 0.06) and lactate (*e.g.*, AUC RA_CE_ 230 ± 108 min*mM *vs.* AR_CE_ 250 ± 109 min*mM, *P* = 0.269, *d* = 0.19) levels were not affected by the order of CE (Fig. 3).

**Table 3:** The heart rate (HR) and a rating of perceived exertion (RPE) in responses to moderate-intensity aerobic exercise (MIAE) and resistance exercise (RE) in study 3.

	RA_CE_	AR_CE_	*P*-value	Effect size
HR, bpm				
MIAE	161 ± 11	159 ± 16	0.455	*d* = 0.18
RE	127 ± 17	136 ± 15	**0.007**	***d* = 0.54**
RPE, 6–20 scale				
MIAE	15 (14–17)	15 (14–16)	0.470	*r* = 0.21
RE	15 (15–17)	16.5 (13–17)	0.784	*r* = 0.08
RPE, CR10 scale				
MIAE	6 (4–8)	5 (4–6)	0.188	*r* = 0.38
RE	7.5 (6–8)	7.5 (6–9)	0.595	*r* = 0.15

Values are mean ± SD or median (IQR). RA_CE_, concurrent exercise (RE + MIAE); AR_CE_, concurrent exercise (MIAE + RE).

Significant values are in bold.

## Discussion

In study 1, we examined the impacts of two bouts of MIAE on blood lactate. Compared with the first bout, the blood lactate response was reduced during the second bout of MIAE. Thus, consistent with our hypothesis, lactate accumulation was reduced in the second bout of AE even at a moderate intensity. Nevertheless, contrary to our hypothesis, blood lactate elevation was not reduced during RE when RE was performed after MIAE as the CE protocol. Similarly, blood lactate elevation during MIAE was not influenced when MIAE was performed after RE. These observations highlighted that the order of the CE trial is not an important consideration for the bioavailability of lactate.

### The impact of the second bout of moderate-intensity aerobic exercise on blood lactate elevation

In the present study, blood glucose was reduced during the second bout of MIAE, implying that gluconeogenesis may not be markedly increased in the second bout of MIAE. Febbraio and Dancey^18^ demonstrated that the muscle glycogen level is associated with MIAE-induced lactate accumulation. In this context, blood lactate is increased during MIAE for 20 min, whereas blood lactate levels can disappear during MIAE at 40 min with muscle glycogen depletion, indicating that the duration of MIAE is a key factor in lactate accumulation^18^. We previously reported similar blood lactate responses to one 40-min bout of MIAE (*i.e.*, continuous MIAE) and two 20-min bouts of MIAE (*i.e.*, repeated MIAE)^23^. Given the results of study 1, the duration of the total exercise period, rather than each bout, may determine blood lactate kinetics when MIAE is separately performed. In other words, AE-induced lactate elevation can be an inaccurate marker of exercise intensity.

During exercise, lactate is distributed as an energy substrate to the brain, heart, and red-oxidative muscle fibers, *etc.*^5, 13–17^. For instance, Rasmussen et al*.*^24^ suggested that lactate metabolism in the human brain during exercise is associated with blood lactate levels and accelerated by blood lactate levels ≥ 2 mM. In the present study 1, the blood lactate level at the end of the first bout of MIAE was 3.4 (2.4–3.9) mM [median (IQR)], and at the end of the second bout of MIAE, it was 2.4 (2.2–3.3) mM (see Fig. 1). In addition to being an energy substrate, in vivo and in vitro studies have shown the positive impact of lactate stimulation on mitochondrial biogenesis in skeletal muscle cells^11^, adipocytes^10^, and the brain^25^. Moreover, the level of blood lactate elevation may play an important role in signaling muscle-brain crosstalk for health^5, 14, 15^. For instance, peroxisome proliferator activated-receptor γ coactivator-1α (PGC-1α) mRNA in skeletal muscle cells is increased by lactate administration in a dose-dependent manner^11^. PGC-1α is not only a transcriptional coactivator of mitochondrial biogenesis but also leads to increased fibronectin type III domain-containing 5 (FNDC5) expression^26^. FNDC5, as irisin, is released into the blood, and circulating irisin can contribute to an increase in brain-derived neurotrophic factor (BDNF)^27, 28^, which is capable of inducing neurogenesis in the brain and is associated with cognitive function^28–30^. Indeed, blood lactate elevation in response to AE can be correlated with exercise-enhanced circulating BDNF^21, 22^. Schiffer et al*.*^31^ demonstrated that circulating BDNF is increased immediately after lactate infusion in humans.

### The impact of concurrent exercise on blood lactate elevation

Inconsistent with our hypothesis based on study 1, in studies 2 and 3, RE-enhanced lactate accumulation was not reduced in the second bout of exercise in the AR_CE_ protocol even though MIAE was performed as the first bout of exercise. In studies 2 and 3, the participants performed cycling exercise as the AE and knee extensions as the RE. Previous studies have indicated that both cycling and knee extensions use mainly lower-limb muscles, such as the vastus lateralis (VL) and rectus femoris^7, 32^, suggesting similar muscle activation. However, based on the degree of lactate elevation, it is assumed that the activation of white-glycolytic muscle fiber (*i.e.*, greater motor-unit recruitment) is more necessary during RE than MIAE. MIAE may not reduce the glycogen in the white-glycolytic fibers, which are used during RE for tetanic contraction. Therefore, the decreased muscle glycogen after MIAE potentially did not affect lactate accumulation in response to RE. Meanwhile, it remains unknown whether high-intensity AE + RE reduces the blood lactate response to RE. Notably, the AUC for lactate was higher in the AR_CE_ trial (*i.e.*, two bouts of exercise) than in the RE trial (*i.e.*, one bout of exercise). From the perspective of lactate bioavailability, CE is a more effective strategy than RE alone.

Creer et al*.*^33^ demonstrated that glycogen content in human VL muscle is decreased by knee extensions consisting of three sets of 10 repetitions at 70% 1-RM with 2-min recovery intervals. Based on this previous finding, it is assumed that RE in the present study reduced muscle glycogen content. Nevertheless, the RA_CE_ trial did not influence the lactate response to MIAE as the second bout of exercise in study 3. Given that the AUC did not differ between the RA_CE_ and AR_CE_ trials, the order of the CE trial is not an important consideration for the bioavailability of lactate. In contrast to two bouts of MIAE (*i.e.*, the same exercise modality), blood lactate levels in the second bout of exercise may not be affected when performing a different type of exercise (*i.e.*, RE and MIAE).

### Perspective

It is well known that ‘exercise is medicine’. In particular, a CE program may offer easy access to health benefits. The results of the present study suggest that the negative impact of the second bout of exercise on lactate accumulation may not occur when a CE program focusing on the lower-limb muscles is performed, regardless of the exercise order. Given that lactate is one of the indicators of the positive impact of exercise on health^5, 10–14^, these observations may partly explain why the CE program is an easy way to improve health.

In contrast to the CE program, the second bout of AE had a reduced blood lactate response even at a moderate intensity, which is the most common AE prescription for health. Lecoultre et al*.*^34^ demonstrated that fructose and glucose coingestion enhances blood lactate elevation during MIAE; in other words, it is possible that energy intake can manipulate lactate kinetics in response to MIAE. To make efficient use of intervals in multiple bouts of the MIAE protocol, a nutritional strategy before performing MIAE may compensate for the lack of lactate accumulation in the multiple bouts of MIAE. Taken together, two bouts of MIAE without energy intake in the interval reduced blood lactate elevation during the second bout of MIAE, which may dampen the positive impact of exercise on some health factors.

### Conclusions

The blood lactate response to a second bout of MIAE was reduced. On the other hand, the blood lactate response to RE was not affected when MIAE was performed before RE, and the blood lactate elevation during the RA_CE_ trial was also at a similar level as that in the AR_CE_ trial. Thus, blood lactate kinetics are not altered by the combined performance of the different types of exercise focusing on the lower-limb muscles (*i.e.*, knee extensions as RE and cycling exercise as AE).

## Methods

### Ethics and participants

All procedures conformed to the *Declaration of Helsinki* and were approved by the Ethics Committee of Ritsumeikan University (BKC-2017-078). Forty-three healthy men participated in the studies after providing written informed consent. All participants were free of neurologic, cardiovascular, or pulmonary disorders, did not take any medication and were nonsmokers. Participants were instructed to avoid strenuous physical activity in the 24 h preceding each experiment visit. Each participant was also asked to abstain from food, alcohol, and caffeine intake for 12 h before each experiment. Experiments were performed at 22–24 °C. Compared with the volume-matched 40-min MIAE, data from study 1 on HR, blood pressure, blood metabolites, cognitive function, and psychological parameters, after only the second bout of the 20-min MIAE, are already published^23^.

### Experimental procedure and trials

For studies 2 and 3, a one-repetition maximum (1-RM) was determined on the first visit to calculate the intensity of bilateral knee extensions at least 7 days before the third and fourth visits. The 1-RM trial was designed using increments of 10 kg until 60–80% of the perceived maximum was achieved. Subsequently, the load was incrementally increased by 1–5 kg until failure, which was indicated by the inability to maintain proper form or complete the repetition. The last acceptable lift with the highest possible load was defined as the 1-RM^7^.

Approximate peak oxygen consumption (VO_2_ peak) was determined to calculate the intensity of cycling the MIAE on the first visit for study 1 and the second visit for studies 2 and 3, at least 4 days before the next visit. The fitness test began at a power of 30 W for 3 min. Subsequently, the workload was increased by 30 W/min until the participants were not able to maintain a cadence of 60 rpm (task failure of a pedaling rate of at least 55 rpm over 5 s despite maximal effort). During the test, breath-by-breath pulmonary gas-exchange data were collected and averaged every 10 s (AE-310S; Minato Medical Science, Japan). Additionally, HR was checked continuously via telemetry (RS400; Polar Electro, Finland). The VO_2_ peak was determined as the highest 30-s value attained prior to exhaustion^8, 19^.

In study 1, twenty participants (age 21 ± 2 years, height 173 ± 3 cm, weight 64 ± 9 kg, and VO_2_ peak 45.9 ± 4.6 ml/kg/min; means ± SDs) performed two identical bouts of the MIAE trial^23^. In study 2, eleven participants (age 22 ± 1 years, height 170 ± 3 cm, weight 62 ± 8 kg, 1-RM 118 ± 21 kg, and VO_2_ peak 47.0 ± 4.3 ml/kg/min) performed two separate trials (RE and AR_CE_ trials) on the third and fourth visits in a randomized, counterbalanced order. In study 3, twelve participants (age 21 ± 2 years, height 172 ± 4 cm, weight 62 ± 6 kg, 1-RM 121 ± 22 kg, and VO_2_ peak 46.9 ± 4.0 ml/kg/min) performed two separate trials (RA_CE_ and AR_CE_ trials) on the third and fourth visits in a randomized, counterbalanced order. For studies 2 and 3, each experimental day was separated by at least 72 h.

**Figure 4:**
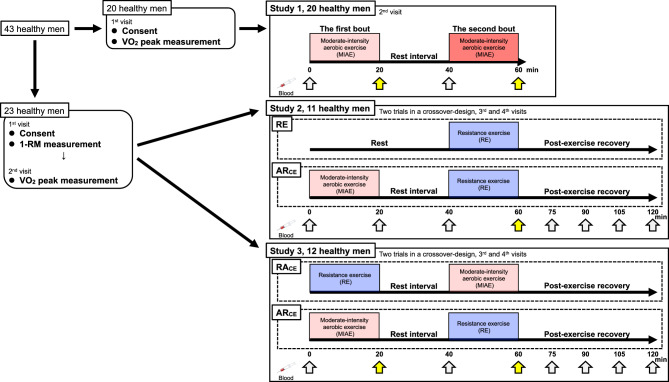
Schematic representation of the experimental design. The yellow arrows indicated the measurement point to compare lactate accumulation in response to each exercise protocol.

In all trials (Fig. 4), every participant attended the laboratory at 0800–1100. Upon arrival, a nurse inserted an 18-gauge cannula in the cephalic vein of the nondominant arm for blood sampling. Afterward, all participants rested in a seated upright position for at least 10 min before data collection began. Blood was collected after measuring HR at baseline. Subsequently, the participants carried out each intervention.

***Study 1*** The participants performed two bouts of MIAE. Before starting the second bout of MIAE, the participants took a 20-min rest interval. The HR, RPE, and blood were collected before and at the end of the first and second bouts.

***Study 2*** In the RE trial, the participants performed RE after a seated upright position for 40-min. In the AR_CE_ trial, the participants performed MIAE and RE with 20 min intervals in a seated upright position. The HR, RPE, and blood were collected before and at the end of each MIAE and RE. To estimate the bioavailability of lactate, blood was collected 4 times at 15-min intervals following RE. At the same time point as the AR_CE_ trial, blood in the RE trial was collected 3 times before the RE trial (*i.e.*, in a seated upright position for 40 min).

***Study 3*** The AR_CE_ trial was performed in the same way as study 2. In the RA_CE_ trial, the participants performed RE and MIAE with 20 min intervals in a seated upright position. The HR, RPE, and blood were collected at the same time point as in study 2.

### Exercise protocols

The MIAE protocol consisted of cycling exercise at an estimated 60% VO_2_ peak (Study 1: 148 ± 16 watts; Study 2: 146 ± 21 watts; Study 3: 148 ± 20 watts) for 20 min^8^.

The RE protocol used bilateral knee extensions at 80% 1-RM (Study 2: 91 ± 13 kg; Study 3: 93 ± 16 kg) and was programmed for 6 sets with 10 repetitions (1-s concentric/1-s eccentric contraction) per set. The participants rested for a 3 min before starting each set of knee extensions^5^; therefore, the total RE protocol time was also 20 min.

### Measurements

The HR was checked via telemetry during each trial (RS400; Polar Electro, Finland).

The psychological response to the MIAE/RE, RPEs for breathing and leg effort were evaluated using the Borg 6–20 scale and the Borg CR10 scale, respectively. The Borg 6–20 scale ranges from 6 (no exertion) to 20 (maximal exertion). The Borg CR10 scale ranges from 0 (nothing at all) to 10 (almost maximum)^35^.

Blood was collected into 1-ml syringes to determine blood glucose and lactate levels, which were measured using glucose (Medisafe FIT Blood Glucose Meter; Terumo, Japan) and lactate analyzers (Lactate Pro 2; Arkray, Japan), respectively.

### Statistical analysis

In the figures, the individual, box-and-whisker, and raincloud plots were created using JASP software (version 0.16.4, University of Amsterdam, Netherlands)^36^. The other data are expressed as the means ± SDs if a normal data distribution was confirmed using the Shapiro‒Wilk test; if not, the data are expressed as medians (IQRs). According to our hypothesis, data at the end of the MIAE/RE were analyzed using a paired *t* test after normal data distribution was confirmed, whereas the Wilcoxon signed-rank test was used if normal data distribution was not confirmed. Similarly, the estimated AUC was also analyzed using a paired *t* test because all AUCs were normally distributed. Statistical significance was indicated by *P* < 0.05. For normal data distribution, Cohen’s *d* effect size using the means and pooled SD were calculated, along with the 95% confidence interval to determine the magnitude of differences. The strength of the effect size of Cohen’s *d* was interpreted as weak (0.20 ≤ *d* < 0.50), medium (0.50 ≤ *d* < 0.80), and large (0.80 ≤ *d*)^37^. For nonnormal data distribution, the effect size, as *r*, was estimated using the *Z* score for the Wilcoxon signed-rank test. The strength of the effect size of *r* was interpreted as weak (0.10 ≤ *r* < 0.30), medium (0.30 ≤ *r* < 0.50), and large (0.50 ≤ *r*)^37^. All statistical analyses were conducted using IBM SPSS software (version 27, Chicago, IL, United States).

## Data Availability

The data that support the findings of this study are available from the corresponding author, H.T., upon reasonable request.
